# Current treatment concepts in implantology in oral and maxillofacial surgery in Germany

**DOI:** 10.1186/s40729-026-00668-4

**Published:** 2026-02-23

**Authors:** Andreas Pabst, Jörg Wiegner, Matthias Schneider, Nils Weyer, Alexander Bartella, Philipp Becker, Alexander-N. Zeller

**Affiliations:** 1 Department of Oral and Maxillofacial Surgery, University Medical Center Mainz, Augustusplatz 2, 55131 Mainz, Germany; 2 Department of Oral and Maxillofacial Surgery, German Armed Forces Central Hospital, Rübenacherstr. 170, 56072 Koblenz, Germany; 3 Private Practice for Oral and Maxillofacial Surgery, Saalstr. 35, 07318 Saalfeld, Germany; 4 Private Practice for Oral and Maxillofacial Surgery, Dr.-Külz-Ring 15, 01067 Dresden, Germany; 5 Private Practice for Oral and Maxillofacial Surgery, Fabrikstr. 10/1, 73728 Esslingen a.N., Germany; 6 Private Practice for Oral and Maxillofacial Surgery, Detmolder Str. 530, 33699 Bielefeld, Germany; 7 Department of Oral and Maxillofacial Surgery, University Medical Center Leipzig, Liebigstr. 12, 04103 Leipzig, Germany; 8 Private Practice for Oral and Maxillofacial Surgery, Theaterstr. 61, 52062 Aachen, Germany; 9 Department of Oral and Maxillofacial Surgery, Hannover Medical School, Carl-Neuberg-Str. 1, 30625 Hannover, Germany

**Keywords:** Oral and maxillofacial surgery, DGMKG, Implantology, Immediate implant placement, Guided surgery, Survey, Germany

## Abstract

**Introduction:**

Dental implantology is a core competency of Oral and Maxillofacial Surgery (OMFS). However, detailed data on the current treatment concepts in implantology in OMFS in Germany are limited. This study analyzed current treatment concepts, clinical practices, decision-making factors, and the adoption of advanced technologies in implantology in OMFS in Germany.

**Material and methods:**

A dynamic online questionnaire with up to 38 questions was sent to 1391 OMFS members of the German Association of Oral and Maxillofacial Surgery (DGMKG). The questionnaire collected general and specific data, such as implantological experience, time points of implant placement (immediate vs. delayed), implant systems and designs, imaging modalities, digital planning, guided surgery, healing, (immediate) prosthetic restorations, follow-up, pre- and postoperative management, and the use of platelet-rich fibrin (PRF). Data analysis was descriptive and anonymous.

**Results:**

276 OMF surgeons participated in the study, with an average of 20 years of experience in implantology. Most worked in private practices without inpatient facilities (66.3%). Most placed 201–500 implants per year (34.78%). 78.99% performed immediate implant placement, mainly to shorten treatment time and preserve alveolar bone. Cone beam CT (88.42%) and panoramic radiographs (68.34%) were the most common imaging modalities. Virtual planning was used by 73.08%, and guided surgery was used by 66.54%, mainly with externally produced guides. Most participants preferred closed implant healing (83.53%). Immediate prosthetic restorations (PR) were rarely performed (57.83% never), and 70.92% did not carry out definitive PR. Risk factors for implant failure were poor oral hygiene (84.72%), limited surgical experience (76.39%), and smoking (75%). PRF was used routinely by 9.72%, and selectively by 45.83%

**Conclusion:**

The results indicate high implantological standards and heterogeneity in current treatment concepts in OMFS implantology in Germany. Possible reasons may include the surgeon’s training and experience, the private practices’ and clinics’ organizational and structural features, and the referral network.

**Clinical relevance:**

This study underscores the importance of implantological education and training, interdisciplinary communication, and the further implementation of guidelines.

**Supplementary Information:**

The online version contains supplementary material available at 10.1186/s40729-026-00668-4.

## Introduction

Dental implants have become a cornerstone of oral rehabilitation and prosthetic restoration for partially or fully edentulous patients, with 10-year survival rates of about 98% for immediate and delayed implant placement [1, 2]. Treatment algorithms in implant surgery are subject to continuous change and adaptation, driven by demographic, surgical, and technological developments.

In elderly patients, dental implants are frequently the only viable option for achieving sufficient prosthetic rehabilitation, and implant-supported prosthetic restorations enable greater oral function than conventional prosthetics [3]. Epidemiological data from the United States demonstrated, along with an overall increase in the prevalence of dental implants from 0.7% to 5.7%, that the most significant absolute increase in implant prevalence (12.9%) occurred among individuals aged 65–74 years between 1999–2000 and 2015–2016 [4]. However, the ongoing change and adoption of implant surgery is influenced not only by demographic shifts. Advances in surgical protocols, such as immediate or early implant placement, biomaterials, digital technologies, and guided surgery, expanded the indications for implants and enabled increasingly individualized treatment concepts [5–8]. Next, artificial intelligence (AI) and virtual reality appear to be relevant aspects that may further increase precision and efficiency in implantology [9, 10].

At the same time, implantology has become more complex, demanding a high level of training, experience, diagnostic and surgical precision, digital literacy, and interdisciplinary coordination. Next, multimorbidity, defined as two or more coexisting conditions in a patient, is globally increasing [11] and poses an increasing challenge in implantology for patient management. Certain systemic conditions or comorbidities may increase the risk of complications associated with dental implants, such as radiotherapy or treatment with high-potency antiresorptive agents (e.g., bisphosphonates) in patients with malignancy [12]. Next, medications such as proton-pump inhibitors or serotonin reuptake inhibitors have been associated with impaired implant osseointegration and subsequent failures [13].

In light of these developments and challenges, Oral and Maxillofacial Surgery (OMFS) plays a central role in implantology due to its surgical experience and ability to manage complex reconstructive and anatomical difficulties, such as severe alveolar ridge atrophy and medically compromised and multimorbid patients. It was shown that over 40% of OMFS trainees in Germany focused on implantology as a subspecialty in their residency [14], underscoring the growing interest of OMFS trainees in this field and reflecting its increasing clinical and educational relevance.

Despite the relevance of implantology in OMFS, detailed data on current treatment concepts, day-to-day clinical practices, decision-making factors, and the adoption of advanced technologies in Germany are limited. The extent to which key aspects such as immediate implant placement, advanced imaging modalities, digital planning and guided surgery, or immediate and definitive implant-supported restorations are implemented in OMFS clinical practice is unclear. Detailed data is essential for identifying trends, unmet needs, challenges, and potential targets for quality improvement, training and education, and guideline development.

This study analyzed current treatment concepts, clinical practices, decision-making factors, and the adoption of advanced technologies in OMFS implantology in Germany.

## Material and methods

This study was designed as a cross-sectional, questionnaire-based survey including OMFS members of the German Association of Oral and Maxillofacial Surgery (DGMKG). A dynamic questionnaire with up to 38 questions and free-text sections (Suppl. material 1) was developed by full-board certified OMF surgeons of the DGMKG. The questionnaire included a broad spectrum of general and specific aspects relevant to implantology. General aspects included demographic and professional background (e.g., type of private practice or clinic setting, years of implantological experience, and implant volume per year). Specific aspects included time points of implant placement (immediate, early, delayed), implant system and design preferences, use of digital planning and guided surgery, pre- and postoperative imaging modalities, pre- and postoperative management, implant stability, healing modalities, immediate and definitive implant-supported prosthetic restorations, follow-up, and platelet-rich fibrin for implant surgery. Most questions were in multiple-choice format with single or multiple answer options, while selected questions included scaled responses and optional free-text sections for further specification. Participants were allowed to skip individual questions. The questionnaire was evaluated internally and externally. External evaluation was performed by full-board certified OMF surgeons with extensive experience in implantology, who were not involved in questionnaire development and in the subsequent data collection. Then, the questionnaire was transferred into the SurveyMonkey® online survey platform (SurveyMonkey® Europe UC, Dublin, Ireland). Another internal evaluation was performed to ensure online survey functionality. Via SurveyMonkey® and the DGMKG mailing list, the DGMKG head office sent an invitation letter with a link to the survey to a total of 2111 OMFS members of the DGMKG. Of these, 566 did not open the invitation, and 142 invitations were rejected, leaving 1391 members who successfully received the survey invitation and the link. The survey ran for eight weeks. Three reminders were sent throughout the run to increase the response rate. Participation was voluntary and anonymous.

The data was transferred to Excel® (Microsoft Corporation, Redmond, WA, USA). Descriptive statistics were used for data analysis. As multiple answers were permitted for some multiple-choice questions, the total number of selected responses may exceed the number of participants who answered that question. The same applies to the sum of the corresponding percentage values, which thus may exceed 100%. Supplemental material 1 overviews the questionnaire, the questions 1–38, the answer options for each question, the number of participants who answered each question, the frequency and the percentage proportion with which each answer of each question was selected, and the answers for the respective free-text sections.

Figures were generated using GraphPad Prism® 9 (GraphPad Software, San Diego, CA, USA). Language and grammar checks, translations and text improvements were conducted using ChatGPT-5® (OpenAI, San Francisco, CA, USA) and Grammarly® (Grammarly Inc., San Francisco, CA, USA).

## Results

### General data

Two hundred seventy-six OMF surgeons participated in the study (response rate 19.84%) with an average of 20 years of professional experience in implantology. Most participants (66.30%, 183/276) reported working in a private practice without inpatient facilities (no own beds or hospital affiliation). A further 15.58% (43/276) indicated working in a private practice with inpatient care (own beds or affiliated hospital beds). A small proportion of participants were based in dental (3.26%, 9/276) or oral surgery private practices (1.09%, 3/276). Medical care centers affiliated with hospitals accounted for 2.17% (6/276) of the responses. Clinicians working in hospitals were represented by 1.09% (3/276) from OMFS sections and 5.07% (14/276) from OMFS departments. University hospitals comprised 5.43% (15/276) of participants.

Regarding the average number of implants placed per year, responses varied widely among participants. The largest group (34.78%, 96/276) reported having placed between 201 and 500 implants. A further 20.29% (56/276) indicated having placed between 501 and 1000 implants, while 19.93% (55/276) had experience with 101 to 200 implants. Smaller proportions reported fewer implant placements: 7.97% (22/276) placed 51–100 implants, 7.61% (21/276) placed 21–50 implants, and 4.71% (13/276) placed 0–20 implants. An additional 4.71% (13/276) reported having placed more than 1000 implants.

### Timepoints of implant placement

**Fig. 1:**
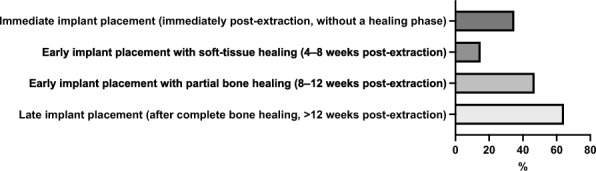
Overview of participants’ preferred time points of implant placement

Figure 1 overviews the preferred time points of implant placement by participants. When asked whether they perform immediate implant placement, the majority of participants (78.99%, 218/276) answered “yes”, while 21.01% (58/276) reported not performing immediate implant placement.

**Fig. 2:**
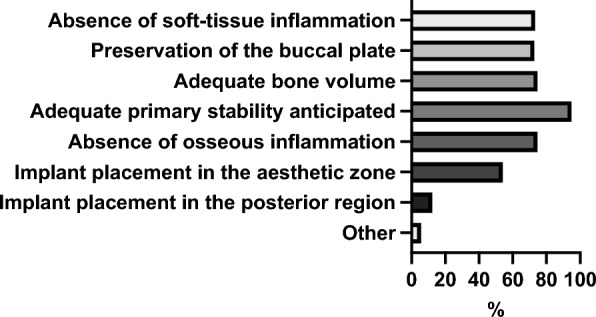
Overview of relevant factors for participants for immediate implant placement

Figure 2 summarizes the factors relevant to participants for immediate implant placement.

Concerning the reasons for immediate implant placement, the most frequently stated motivation was a faster overall treatment process from tooth extraction to definitive prosthetic restoration (70.09%, 150/214). Prevention or reduction of alveolar ridge resorption and avoidance of additional augmentation procedures were also commonly mentioned (62.62%, 134/214). More than half of the participants (55.61%, 119/214) cited improved aesthetic outcomes (e.g., pink–white aesthetics) as an essential factor. Further motivations included patient or referrer preference (49.53%, 106/214), the possibility of an immediate (temporary) implant-supported prosthetic restoration (27.10%, 58/214), and economic considerations such as higher efficiency or productivity (7.48%, 16/214).

Among the 58 respondents who reported not performing immediate implant placement, the most frequently cited reason was the perceived high risk of implant loss or failure (58.62%, 34/58). Lack of experience or expertise (24.14%, 14/58) and previous negative experiences (22.41%, 13/58) were also common responses. Additional factors included insufficient demand from referrers or patients (18.97%, 11/58), perceived lack of benefit (15.52%, 9/58), limited time resources (6.90%, 4/58), and high associated costs (6.90%, 4/58). Only a small number of participants (3.45%, 2/58) cited missing structural or personnel resources as the main reason.

### Implant systems and designs

Table 1 summarizes the attributes of implant designs that participants routinely use. Table 2 summarizes general factors relevant to participants in selecting the implant systems and designs used. Table 3 summarizes the surgical factors relevant to participants’ choice of implant systems and designs.

**Table 1:** Which attributes best describe the implant designs you routinely use? (multiple answers possible)

Answer choices	Responses	
Conical implant (conical–conical)	40.15%	104
Cylindrical implant (parallel-walled)	40.15%	104
Tapered implant (combined conical–cylindrical)	60.62%	157
Tissue-level implant	37.07%	96
Bone-level implant	88.80%	230
Short implant (e.g. < 8 mm)	20.85%	54
Diameter-reduced implant (e.g. < 3 mm)	9.65%	25
Ceramic implant (e.g. zirconia)	17.37%	45
Other (please specify)	3.47%	9

**Table 2:** Which general factors are decisive for you when selecting the implant system/design? (multiple answers possible)

Answer choices	Responses	
Personal experience / expertise	86.54%	225
Patient preference	11.92%	31
Referrer preference	69.62%	181
Practice / organizational structure	20.00%	52
Potential risk of complications	24.62%	64
Scientific evidence / literature	33.46%	87
Costs	17.31%	45
Biological and mechanical safety	47.31%	123
Manufacturer customer service	32.69%	85
Traditional reasons (“we have always used it”)	11.92%	31
Reported patient intolerances	8.46%	22
Allergy testing	4.23%	11
None	0.77%	2
Other (please specify)	2.69%	7

**Table 3:** Which surgical factors are decisive for you when selecting the implant system/design? (multiple answers possible)

Answer choices	Responses	
Anatomical region and available bone volume	75.00%	195
Bone quality	58.46%	152
Timing of implant placement (e.g. immediate vs. delayed placement)	47.69%	124
Potential risk of complications related to implant design	29.62%	77
Soft-tissue conditions	37.31%	97
Primary stability	50.77%	132
Aesthetics	41.54%	108
Prosthetic planning	39.62%	103
None	9.62%	25
Other (please specify)	3.46%	9

42.80% of participants (110/257) reported using diameter-reduced implants in cases of limited space in the anterior region. Diameter-reduced implants were also used for minimally invasive treatment approaches in elderly patients by 19.84% (51/257) of participants, and for narrow alveolar ridges by 10.51% (27/257). A smaller proportion (5.84%, 15/257) indicated using diameter-reduced implants specifically to avoid augmentation procedures. Conversely, 42.80% (110/257) stated that they do not use diameter-reduced implants.

59.07% of the participants (153/259) reported using short implants in cases with reduced vertical bone height. Short implants were also used to avoid augmentation procedures by 31.66% (82/259), particularly in the posterior mandible (35.52%, 92/259) and, to a lesser extent, in the posterior maxilla (12.74%, 33/259). A further 21.24% (55/259) indicated that they used short implants in minimally invasive treatment concepts for elderly patients, while 24.32% (63/259) stated that they do not use short implants.

### Preoperative imaging modalities, digital planning, and guided surgery

Cone beam computed tomography (CBCT) was the most frequently used imaging modality before implant placement, reported by 88.42% (229/259) of participants. Panoramic radiographs were also commonly used (68.34%, 177/259), while intraoral radiographs were used by 10.42% (27/259). Computed tomography (CT) was utilized by 3.86% (10/259), and intraoral scanners by 19.69% (51/259). Less frequently mentioned were face scanners (0.77%, 2/259), lateral cephalometric radiographs (0.39%, 1/259), nasal sinus radiographs (0.39%, 1/259), and magnetic resonance imaging (MRI) (0.39%, 1/259). Only one participant (0.39%, 1/259) reported not using any imaging method.

190 participants out of 260 (73.08%) reported using virtual planning tools in implantology, at least occasionally. In contrast, 26.92% (70/260) stated that they do not utilize virtual planning in their workflow.

44.09% of the participants (82/186) reported using virtual planning only in complex cases, such as those with limited bone height or width, difficult anatomical conditions, or the need for precise implant parallelism. A further 25.27% (47/186) stated that they routinely use virtual planning for all implantations, and 22.04% (41/186) use it for most implant cases. Virtual planning was also applied during augmentation before subsequent implantation by 15.05% (28/186), and in 17.20% (32/186) of cases involving simultaneous implantation and augmentation. Aesthetic considerations played a role for 28.49% (53/186) of participants, particularly in the anterior region. Only 16.67% (31/186) reported using virtual planning solely in exceptional cases, while none stated that they never use it.

The vast majority of participants (89.30%, 167/187) reported that they personally carry out virtual implant planning. In 10.16% (19/187) of cases, planning was performed by an assistant dentist, and in 17.65% (33/187) by a dental technician. External template manufacturers or planning services were involved in 12.83% (24/187) of cases, and referring colleagues (e.g., general dentists or orthodontists) in 4.28% (8/187). None indicated that dental auxiliary staff were responsible for the planning process.

Among the 70 participants who reported not using virtual planning, the most frequently cited reasons were high costs (61.43%, 43/70) and excessive time requirements (55.71%, 39/70). Almost half of the participants (48.57%, 34/70) reported seeing no benefit in using virtual planning. A lack of demand from referrers or patients was mentioned by 41.43% (29/70), and 35.71% (25/70) stated insufficient experience or expertise as a barrier. Additional factors included a lack of personnel or structural resources (11.43%, 8/70) and negative past experiences (5.71%, 4/70).

66.54% of the participants (171/257) reported using guided surgery techniques, at least occasionally. In contrast, 33.46% (86/257) stated that they do not use guided surgery in their implantological practice.

**Fig. 3:**
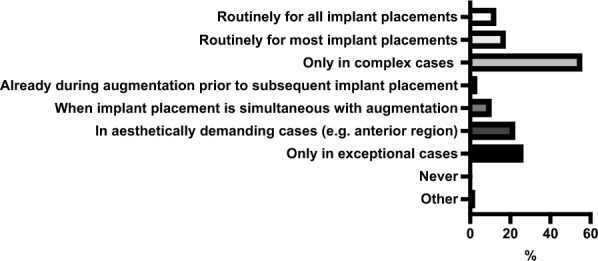
Overview of the frequency of the use of guided surgery (template-based procedures) for implant surgery by participants

Figure 3 overviews the frequency with which participants use guided surgery (template-based procedures) for implantological interventions.

The majority of the participants (68.05%, 115/169) reported that an external dental laboratory produces their surgical guides. External service providers such as “Dedicam” or “Magelan” were used by 22.49% (38/169) of participants. In-house production was less common: 21.89% (37/169) printed their guides in-house within the practice, 3.55% (6/169) milled them in-house, and 8.88% (15/169) manufactured them using conventional methods.

**Fig. 4:**
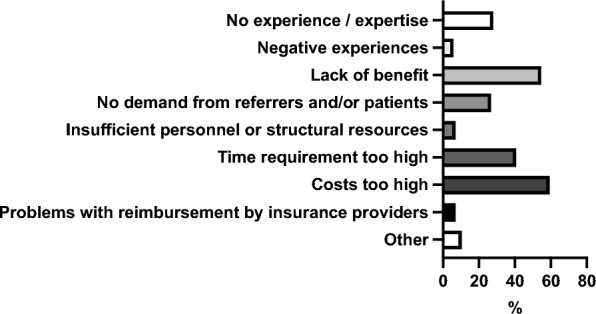
Overview of participants’ reasons for not using guided surgery (template-based procedures) for implant surgery

Figure 4 overviews the reasons why participants are not using guided surgery (template-based procedures) for implantological interventions.

### Implant stability, healing modalities, and postoperative imaging modalities

Manual inspection and clinical assessment were the most commonly used methods for evaluating primary implant stability by most participants (77.69%, 195/251). Percussion testing was also frequently employed (51.39%, 129/251), followed by insertion torque measurement (39.04%, 98/251). Radiographic methods were applied by a smaller proportion of participants, including panoramic radiographs (58.96%, 148/251) and intraoral radiographs (36.65%, 92/251), while CBCT was used by 5.18% (13/251). Resonance frequency analysis devices such as “Osstell” or “Neotell” were used by 15.14% (38/251). Only a small minority reported not performing any assessment (2.79%, 7/251) or indicated other unspecified methods (2.79%, 7/251).

A clear majority of participants (83.53%, 208/249) stated that they prefer closed healing of dental implants when given the choice based on stability and clinical circumstances. In contrast, 16.47% (41/249) favored open healing.

Table 4 summarizes the reasons for the participants to prefer closed implant healing.

**Table 4:** For what reasons or in which scenarios would you prefer closed (submerged) implant healing? (multiple answers possible)

Answer choices	Responses	
Improved wound healing through primary wound closure	68.80%	172
Lower risk of postoperative infection	65.60%	164
Less mechanical loading of the implant during the healing phase	60.80%	152
Financial reasons (second-stage surgery)	2.80%	7
Simultaneous possibility of augmentation procedures	74.80%	187
In patients with an increased risk of complications (e.g. smoking, diabetes, immunosuppression, etc.)	64.80%	162
Following prior augmentation	35.60%	89
None	1.20%	3
Other (please specify)	3.60%	9

Table 5 summarizes the reasons for the participants to prefer open implant healing.

**Table 5:** For what reasons or in which scenarios would you prefer open (non-submerged) implant healing? (multiple answers possible)

Answer choices	Responses	
Possibility of immediate provisional prosthetic restoration	39.84%	100
Improved soft-tissue management	40.24%	101
Lower risk of postoperative infection	6.37%	16
Avoidance of a second surgical procedure for implant exposure	51.00%	128
Less postoperative swelling	8.37%	21
Earlier access for prosthetic procedures	19.12%	48
Reduction of total treatment time	27.09%	68
Financial reasons (reduction of costs)	15.14%	38
None	17.93%	45
Other (please specify)	4.78%	12

Panoramic radiography was by far the most commonly used imaging method after implant placement, reported by 94.82% (238/251). Intraoral radiography was used by 41.43% (104/251), while CBCT was used by 9.56% (24/251). Other modalities, such as intraoral scanning (3.19%, 8/251), face scanning (0.40%, 1/251), and sinus imaging (0.40%, 1/251), played only a minor role. A small proportion of respondents (0.40%, 1/251) reported performing no radiographic imaging after implant placement.

### Immediate prosthetic restorations, follow-up, and definitive prosthetic restorations

Regarding immediate implant-supported provisional prosthetic restorations, 57.83% of participants (144/249) stated they never use this procedure. A further 38.55% (96/249) reported applying it in up to 20% of their cases, while only a small minority (2.41%, 6/249) reported more frequent use of 21% to 40% of cases. In total, 1.20% (3/249) reported performing this in 41% to 100% of their cases.

**Fig. 5:**
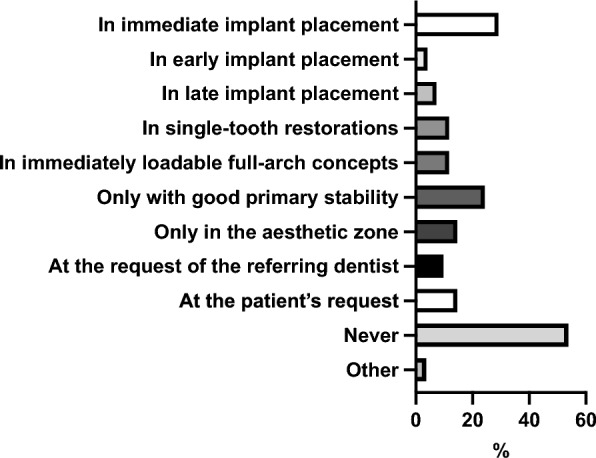
Overview of the cases in which participants perform immediate implant-supported provisional prosthetic restoration after implant placement

Figure 5 overviews the cases in which participants perform immediate implant-supported provisional prosthetic restoration after implant placement.

48% of the participants (120/250) reported that all of their implant patients are routinely invited to follow-up (recall) appointments. Another 27.2% (68/250) indicated that only selected patients are included in recall programs. In contrast, 24.8% (62/250) stated that they do not conduct any systematic recall for their implant patients.

Most participants (70.92%, 178/251) reported that they do not perform definitive implant-supported prosthetic restorations themselves. 24.7% (62/251) indicated that they do so occasionally, while only 4.38% (11/251) stated that they always carry out the prosthetic phase personally.

Table 6 overviews the reasons why participants do not perform definitive implant-supported prosthetic restorations themselves.

**Table 6:** Why do you not personally perform definitive implant-supported prosthetic restorations in addition to implant surgery? (multiple answers possible)

Answer choices	Responses	
No experience / expertise	31.64%	56
Negative experiences	0.56%	1
No personal interest	28.81%	51
No financial benefit	2.26%	4
Performed by another colleague in the practice/clinic	13.56%	24
No demand from referrers and/or patients	26.55%	47
Insufficient personnel or structural resources	9.60%	17
Time requirement too high	5.08%	9
Costs too high	0.00%	0
Work in a purely surgical referral practice	77.40%	137
Other (please specify)	3.95%	7

### Pre- and postoperative management for implant surgery

Tables 7and 8 summarize results concerning the pre- and postoperative management for implant surgery used by participants.

**Table 7:** Which preoperative management do you routinely use for implant placement? (multiple answers possible)

Answer choices	Responses	
Pre-emptive analgesia (e.g. ibuprofen)	15.71%	11
Glucocorticoids (e.g. dexamethasone) oral	1.43%	1
Glucocorticoids (e.g. dexamethasone) intravenous	4.29%	3
Antibiotics oral	72.86%	51
Antibiotics intravenous	10.00%	7
Antiseptic rinses (e.g. chlorhexidine)	81.43%	57
Professional dental cleaning	52.86%	37
Optimization of patient compliance	32.86%	23
None of the above	2.86%	2
Other (please specify)	4.29%	3

**Table 8:** Which postoperative management do you use after implant placement? (multiple answers possible)

Answer choices	Responses	
Pain medication as needed	68.06%	49
Pain medication with a medication plan	27.78%	20
Glucocorticoids (e.g. dexamethasone) oral	4.17%	3
Glucocorticoids (e.g. dexamethasone) intravenous	4.17%	3
Antibiotics oral	76.39%	55
Antibiotics intravenous	2.78%	2
Antiseptic rinses (e.g. chlorhexidine)	59.72%	43
Cooling	76.39%	55
Restriction of physical activity / avoidance of sports	56.94%	41
Soft diet / dietary modification	80.56%	58
Temporary avoidance of dairy products	12.50%	9
None	0.00%	0
Other (please specify)	0.00%	0

**Fig. 6:**
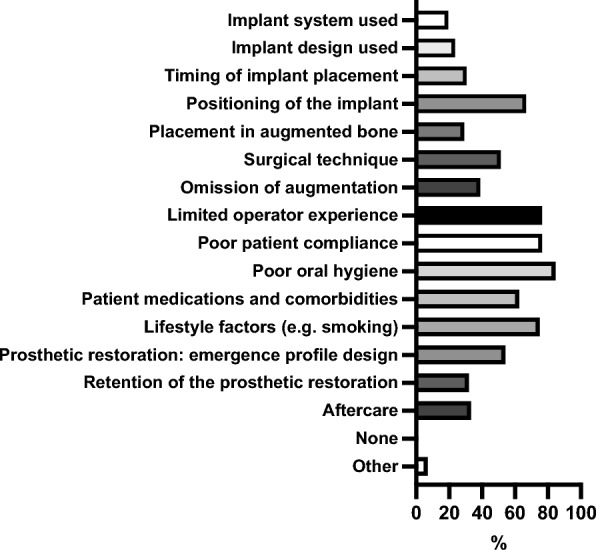
Overview of factors negatively influencing the long-term outcome of implants by participants

Figure 6 overviews the factors negatively influencing the long-term outcome of implants by participants.

The factors most frequently regarded as absolute contraindications for implant placement by participants were radiotherapy within the previous 12 months before planned implantation (66.20%, 47/71) and treatment with high-potency antiresorptive agents, such as bisphosphonates used in breast cancer therapy (57.75%, 41/71). Poor patient compliance (47.89%, 34/71) and poor oral hygiene (40.85%, 29/71) were also frequently cited as absolute contraindications. Other conditions considered absolute contraindications by some participants included therapy with RANK-L inhibitors such as denosumab (29.58%, 21/71), radiotherapy more than 12 months before implantation (14.08%, 10/71), and the use of low-potency antiresorptive agents (e.g., bisphosphonates for osteoporosis) (9.86%, 7/71). Systemic diseases such as diabetes mellitus, the use of proton pump inhibitors, or antidepressant medication were rarely mentioned (each 1.41%, 1/71). Lifestyle factors such as smoking were identified by 4.23% (3/71) as absolute contraindications. A small proportion of participants (9.86%, 7/71) indicated that none of the listed factors represented absolute contraindications to implant placement in their practice.

### Use of platelet-rich fibrin for implant surgery

Some participants (9.72%, 7/72) reported using platelet-rich fibrin (PRF) in all implant cases, while 45.83% (33/72) reported using it selectively in specific situations. In contrast, 44.44% (32/72) reported not using PRF at all in their implantological practice.

The most commonly reported use of PRF was for the biologization of biomaterials such as bone grafts and membranes (63.59%, 117/184), followed by its application into the surgical site (58.15%, 107/184). Other frequently mentioned uses included introducing PRF into the implant bed (20.65%, 38/184), biologizing the implant surface (20.11%, 37/184), and covering exposed implant components (18.48%, 34/184). Injection of liquid PRF into the surgical site was reported less frequently (15.76%, 29/184). Additionally, 27.17% (50/184) of respondents provided other comments or specified additional indications.

Supplemental material 1 overviews the questionnaire, the questions 1–38, the answer options for each question, the number of participants who answered each question, the frequency and the percentage proportion with which each answer of each question was selected, and the answers for the respective free-text sections.

## Discussion

This study analyzed current treatment concepts, clinical practices, decision-making factors, and the adoption of advanced technologies in implantology in Germany by OMF surgeons. The results reveal considerable heterogeneity in different treatment concepts in implantology, attributable to a range of underlying factors. In Germany, OMF surgeons perform implant surgery in various settings, such as (non-) university hospitals, private practices (with and without inpatient facilities), and hospital-based outpatient centers. These differing institutional frameworks entail variations in patient populations, referral frameworks, organizational structures, clinical workflows, and exposure to digital or interdisciplinary treatment concepts. The lack for inpatient treatment could be particularly relevant in the clinical decision-making process. This effect is also recognized in other dental specialties, such as prosthodontics, where structural factors, such as the healthcare and insurance system, have been shown to influence clinical decision-making [15]. Next, the individual education, training, and surgical experience of each OMF surgeon represent relevant factors, as they influence clinical decision-making and procedural preferences. The influence of education and training on clinical decision-making is known and can already be seen among undergraduate dental students, where differences in training directly impact their clinical decision-making [16].

Regarding the time point of implant placement, most participants (about 80%) reported performing immediate implant placement, primarily because of the associated reduction in overall treatment time. The primary reason cited for not performing immediate implant placement was the perceived high risk of implant loss or failure. A recent systematic review and meta-analysis demonstrated comparable implant survival rates for both immediate and delayed implant placement. However, immediately placed implants appear to carry a slightly higher risk of failure, with reported survival rates of approximately 90–95%, compared to rates exceeding 95% for delayed placement [17]. Even in periapical lesions, immediate implant survival rates are reported to be comparable to those in healthy bone [18]. Other studies reported 2-year survival rates of 98.4% for immediate-placed implants [19] and 98.4% vs. 98.6% for immediate- and delayed-placed implants [1]. Overall, survival rates of immediately and delayed placed implants are comparable. Many participants perform early implant placement after 8–12 weeks, which can offer a balance between time efficiency and treatment safety. A current topic in immediate implant placement concerns whether the peri-implant osseous gap should be filled, e.g., with bone grafts or substitutes, and whether a simultaneous soft tissue graft should be performed [20]. These two aspects may also be of particular interest, as many participants of the current study consider primary stability a prerequisite for immediate implant placement. In this context, it is unclear whether gap filling increases primary implant stability. A study could not find differences in implant stability comparing PRF, xenogeneic and allogeneic bone substitute for gap filling at time point of surgery and follow up [21]. Furthermore, digital technologies, such as dynamic navigation and robotic computer-assisted implant surgery, are expected to gain increasing importance in the context of immediate and early implant placement [22]. Next, immediate loading, particularly in the anterior region, is gaining increasing relevance. Moreover, integrating digital workflows, such as digital prosthetic planning, appears to offer distinct advantages over conventional prosthetics [23].

More than 40% of the participants reported not using diameter-reduced implants, despite a continuously increasing clinical reliability [24]. One possible explanation could be the surgeons’ preference, based on their surgical expertise, for augmentation procedures and the use of wider implants.

Preoperative imaging modalities, CBCT was the most preferred technique among participants. A possible explanation for this preference is the high frequency of digital planning and guided surgery, for which preoperative CBCT imaging is essential. This phenomenon has also been demonstrated in a previous study and is further confirmed by the present findings [25]. The enhancement of surgical precision achieved through CBCT, digital planning, and guided surgery is beyond doubt [26]. Overall, CBCT use must remain justified according to the ALADA principle, particularly when both pre- and postoperative CBCT is performed. In this study, most participants used panoramic radiographs for postoperative imaging. The benefit of digital planning and guided surgery may vary with surgeons’ experience. It may substantially reduce errors among less experienced surgeons, while its relative advantage for experts may be limited. In the future, MRI scans may be more important in implant surgery since it can avoid radiation exposure. MRI was reported to be sufficient for digital planning and guided surgery with comparable results to CBCT [27, 28]. Data is insufficient to evaluate MRI for implant surgery in detail.

Most participants (> 50%) reported that they never perform immediate implant-supported provisional restorations. In cases where such procedures are carried out, they are typically limited, e.g., to situations involving immediate implant placement and adequate primary stability. Implant survival rates of immediately loaded implants are different, ranging from 94.4–100% in the toothless mandible, 85.7–100% in single implants, and 95.5–100% toothless jaws and fixed prosthesis [29]. Over 70% of participants never perform immediate implant-supported provisional prosthetic restorations. This may be attributable to the referral structure, as referring dentists often prefer to perform the prosthetic restoration themselves. On the other hand, particularly in the context of immediate implant placement, this approach could lead to a significantly reduced overall treatment time, considering that under normal circumstances, implant healing and osseointegration typically require between 3 and 8 months [30].

In the pre- and postoperative management of implant surgery, oral antibiotics are frequently prescribed by participants. However, their routine use in implantology remains controversial. A recent systematic review and meta-analysis found no significant benefit of antibiotics in reducing implant failure or postoperative complications among healthy patients [31], findings also supported by other studies [32]. The four most frequently cited factors associated with an increased risk of implant failure were poor oral hygiene, limited patient compliance, lifestyle factors, and the surgeon’s experience. Additionally, patient medications and systemic comorbidities were often mentioned. These findings underscore the patient selection and individualized risk assessment in OMFS implantology. This may be explained by the fact that OMF surgeons frequently receive referrals for more complex implant cases or for patients presenting with pre-existing conditions and risk factors. Also, implant positioning was a relevant factor for failure risk. This may explain participants’ frequent use of digital planning and guided surgery, as these approaches can enhance precision in complex or high-risk implant cases [33].

Over 50% of participants reported using PRF selectively or in all cases for implant surgery, most of them for biologization of biomaterials and injection into the surgical site. In addition to improved wound healing, a reason for PRF injection into the surgical site may be reduced pain levels [34]. Some participants reported using PRF for implant surface biologization, which may increase implant stability [35]. Even compared to data from a previous study, PRF is a well-established and frequently used option in implant and augmentative surgery [25].

This study has several limitations. The majority of participants (> 80%) reported working in private practices, with or without inpatient facilities, which may have introduced observation bias, further compounded by a response rate of approximately 20%. The responding rate is in a range demonstrated in previous studies [25, 36, 37]. Moreover, surveys on implantology are predominantly completed by individuals with a particular interest in or engagement in this field, potentially leading to self-selection bias. Conversely, the strengths of this study include the relatively large overall sample size and the high level of professional experience among respondents, both of which enhance the validity and reliability of the findings. A further study must include quality indicators in dental implantology based on the three main levels of quality management, specifically structure and process quality and patient outcome and compare these data with general data and specific data, e.g., treatment concepts in implantology [38].

## Conclusions

There is noticeable heterogeneity in current treatment concepts in OMFS implantology in Germany, e.g., regarding the timing of implant placement and the imaging modalities used. Possible reasons include the surgeon’s training and experience, the private practices’ and clinics’ organizational, structural, and personal features, and the referral network. A clear trend toward the implementation of digital technologies in implantology performed by OMF surgeons is evident, encompassing methods such as digital planning and guided surgery. Consequently, digital literacy is becoming an increasingly critical competency in OMFS, complementing the need for comprehensive implantological education and training, interdisciplinary communication, and the continued implementation of evidence-based guidelines.

## Supplementary Information

Additional file 1.

## Data Availability

The data are available upon reasonable request.
