# Supplementary material for: Current treatment concepts in implantology in oral and maxillofacial surgery in Germany
Source: Int J Implant Dent. 2026 Feb 23;12:9. doi: 10.1186/s40729-026-00668-4 (PMC12929735; doi:10.1186/s40729-026-00668-4)
Supplement: Supplementary file 1 — Additional file 1. [file 40729_2026_668_MOESM1_ESM.docx]

| 1. In which type of facility are you primarily practicing implantology? |  |  | |  |  |  |
| --- | --- | --- | --- | --- | --- | --- |
| Answer choices | Responses | | |  |  |  |
| Referral practice without inpatient care (no in-house or affiliated beds) | 66.30% | 183 | |  |  |  |
| Referral practice with inpatient care (own beds / affiliated beds) | 15.58% | 43 | |  |  |  |
| Dental practice | 3.26% | 9 | |  |  |  |
| Oral surgery practice | 1.09% | 3 | |  |  |  |
| Hospital-affiliated medical care center (MVZ) | 2.17% | 6 | |  |  |  |
| Hospital (OMFS section) | 1.09% | 3 | |  |  |  |
| Hospital (OMFS main department) | 5.07% | 14 | |  |  |  |
| University hospital | 5.43% | 15 | |  |  |  |
|  | Answered | 276 | |  |  |  |
|  | Skipped | 0 | |  |  |  |
|  |  |  | |  |  |  |
| 2. How many years have you been practicing implantology? |  |  | |  |  |  |
| Answer Choices | Average Number |  | |  |  |  |
| years | 20.30 |  | |  |  |  |
|  |  |  | |  |  |  |
| 3. How many implants do you currently place yourself per year? |  |  | |  |  |  |
| Answer Choices | Responses | | |  |  |  |
| 0–20 | 4.71% | 13 | |  |  |  |
| 21–50 | 7.61% | 21 | |  |  |  |
| 51–100 | 7.97% | 22 | |  |  |  |
| 101–200 | 19.93% | 55 | |  |  |  |
| 201–500 | 34.78% | 96 | |  |  |  |
| 501–1000 | 20.29% | 56 | |  |  |  |
| >1000 | 4.71% | 13 | |  |  |  |
|  | Answered | 276 | |  |  |  |
|  | Skipped | 0 | |  |  |  |
|  |  |  | |  |  |  |
| 4. At which time points do you prefer to perform implant placement? (multiple answers possible) |  | |  |  |  |  |
| Answer choices | Responses | | |  |  |  |
| Immediate implant placement (immediately after tooth extraction, without a healing phase) | 34.91% | 96 | |  |  |  |
| Early implant placement with soft-tissue healing (4–8 weeks after extraction) | 14.91% | 41 | |  |  |  |
| Early implant placement with partial bone healing (8–12 weeks after extraction) | 46.91% | 129 | |  |  |  |
| Late implant placement (after complete bone healing, >12 weeks after extraction) | 64.36% | 177 | |  |  |  |
|  | Answered | 275 | |  |  |  |
|  | Skipped | 1 | |  |  |  |
|  |  |  | |  |  |  |
| 5. Do you perform immediate implant placement? |  |  | |  |  |  |
| Answer choices | Responses | | |  |  |  |
| YES | 78.99% | 218 | |  |  |  |
| NO | 21.01% | 58 | |  |  |  |
|  | Answered | 276 | |  |  |  |
|  | Skipped | 0 | |  |  |  |
|  |  |  | |  |  |  |
| 6. Which factors, in your view, are necessary for or indicate immediate implant placement? (multiple answers possible) |  |  |  |  |  |  |
| Answer choices | Responses | | |  |  |  |
| Adequate primary stability anticipated | 94.84% | 202 | |  |  |  |
| Adequate bone volume | 74.65% | 159 | |  |  |  |
| Preservation of the buccal plate | 72.77% | 155 | |  |  |  |
| Absence of soft-tissue inflammation | 73.24% | 156 | |  |  |  |
| Absence of osseous inflammation | 74.65% | 159 | |  |  |  |
| Implant placement in the aesthetic zone | 53.99% | 115 | |  |  |  |
| Implant placement in the posterior region | 12.21% | 26 | |  |  |  |
| Other (please specify):  healthy patient  no antiresorptive therapy; apical bone gain required  thick gingival phenotype  patient compliance  single-rooted tooth  guided surgery  non-smoker  patient preference, patient age  non-smoker  following dental trauma (e.g. fracture)  only in the anterior region  good patient compliance | 5.63% | 12 | |  |  |  |
|  | Answered | 213 | |  |  |  |
|  | Skipped | 63 | |  |  |  |
|  |  |  | |  |  |  |
| 7. Why do you perform immediate implant placement? (multiple answers possible) |  |  | |  |  |  |
| Answer choices | Responses | | |  |  |  |
| Faster treatment process (from extraction to definitive prosthetic restoration) | 70.09% | 150 | |  |  |  |
| Reduction or avoidance of alveolar ridge atrophy and potential avoidance of augmentation | 62.62% | 134 | |  |  |  |
| Improved aesthetic outcome (e.g., pink–white esthetics) | 55.61% | 119 | |  |  |  |
| Economic reasons (e.g., increased revenue / time efficiency) | 7.48% | 16 | |  |  |  |
| Referrer and/or patient preference | 49.53% | 106 | |  |  |  |
| Possibility of immediate (temporary) implant-supported prosthetic restoration | 27.10% | 58 | |  |  |  |
| Other (please specify):  fixed immediate restoration in the aesthetic zone  avoidance of further surgical interventions, especially in elderly and multimorbid patients  uncomplicated treatment courses; economic considerations  All-on-x | 1.87% | 4 | |  |  |  |
|  | Answered | 214 | |  |  |  |
|  | Skipped | 62 | |  |  |  |
|  |  |  | |  |  |  |
| 8. Why do you not perform immediate implant placement? (multiple answers possible) |  |  | |  |  |  |
| Answer choices | Responses | | |  |  |  |
| No experience / expertise | 24.14% | | | 14 | | |
| Negative experiences | 22.41% | 13 | |  |  |  |
| Lack of benefit | 15.52% | 9 | |  |  |  |
| No demand from referrers and/or patients | 18.97% | 11 | |  |  |  |
| Insufficient personnel or structural resources | 3.45% | 2 | |  |  |  |
| Time requirement too high | 6.90% | 4 | |  |  |  |
| Costs too high | 6.90% | 4 | |  |  |  |
| Risk of implant loss or failure too high | 58.62% | 34 | |  |  |  |
| Other (please specify):  implant placement in reconstructed jaws using microvascular bone grafts  poor statistics  not practicing implantology  patients’ expectations regarding the success of immediate implant placement  tooth loss is generally a process that develops over decades; biological processes require time and cannot be accelerated; these are purely elective procedures, and “immediate” is at best a marketing concept, therefore financially motivated  high-risk patients  time management  not offered in our clinic due to the patient population (often tumor patients with a history of radiotherapy)  planned for the future  patients do not present at the optimal time; they are initially pre-treated by general dentists; therefore, wound revision with socket preservation is often performed followed by delayed implant insertion  fear of failure | 18.97% | 11 | |  |  |  |
|  | Answered | 58 | |  |  |  |
|  | Skipped | 218 | |  |  |  |
|  |  |  | |  |  |  |
|  |  |  | |  |  |  |
| 9. Which attributes best describe the implant designs you routinely use? (multiple answers possible) |  | | |  | |  |
| Answer choices | Responses | | |  | | |
| Conical implant (conical–conical) | 40.15% | 104 | |  |  |  |
| Cylindrical implant (parallel-walled) | 40.15% | 104 | |  |  |  |
| Tapered implant (combined conical–cylindrical) | 60.62% | 157 | |  |  |  |
| Tissue-level implant | 37.07% | 96 | |  |  |  |
| Bone-level implant | 88.80% | 230 | |  |  |  |
| Short implant (e.g. <8 mm) | 20.85% | 54 | |  |  |  |
| Diameter-reduced implant (e.g. <3 mm) | 9.65% | 25 | |  |  |  |
| Ceramic implant (e.g. zirconia) | 17.37% | 45 | |  |  |  |
| Other (please specify):  Straumann SP and BLT  not practicing implantology  conical internal connection; provisional abutments  I do not place implants  9  hybrid implants  unusual connection between implant and abutment  Roxolid preferred  . | 3.47% | 9 | |  |  |  |
|  | Answered | 259 | |  |  |  |
|  | Skipped | 17 | |  |  |  |
|  |  |  | |  |  |  |
| 10. Which general factors are decisive for you when selecting the implant system/design? (multiple answers possible) |  | | |  | |  |
| Answer choices | Responses | | |  | | |
| Personal experience / expertise | 86.54% | 225 | |  |  |  |
| Patient preference | 11.92% | 31 | |  |  |  |
| Referrer preference | 69.62% | 181 | |  |  |  |
| Practice / organizational structure | 20.00% | 52 | |  |  |  |
| Potential risk of complications | 24.62% | 64 | |  |  |  |
| Scientific evidence / literature | 33.46% | 87 | |  |  |  |
| Costs | 17.31% | 45 | |  |  |  |
| Biological and mechanical safety | 47.31% | 123 | |  |  |  |
| Manufacturer customer service | 32.69% | 85 | |  |  |  |
| Traditional reasons (“we have always used it”) | 11.92% | 31 | |  |  |  |
| Reported patient intolerances | 8.46% | 22 | |  |  |  |
| Allergy testing | 4.23% | 11 | |  |  |  |
| None | 0.77% | 2 | |  |  |  |
| Other (please specify):  not practicing implantology  two-piece system  long-term success of peri-implant bone preservation  manufacturer’s headquarters location (e.g. Basel for Camlog)  established companies carry a lower risk of financial instability; therefore, future availability for adjustments or replacements is more likely  I only own the one surgical kit from one company; I had to pay for most of the kit myself. I own two kits. I had to purchase all screwdrivers myself.  stable internal connection to the abutment | 2.69% | 7 | |  |  |  |
|  | Answered | 260 | |  |  |  |
|  | Skipped | 16 | |  |  |  |
|  |  |  | |  |  |  |
| 11. Which surgical factors are decisive for you when selecting the implant system/design? (multiple answers possible) |  | | |  | |  |
| Answer choices | Responses | | |  | | |
| Anatomical region and available bone volume | 75.00% | 195 | |  |  |  |
| Bone quality | 58.46% | 152 | |  |  |  |
| Timing of implant placement (e.g. immediate vs. delayed placement) | 47.69% | 124 | |  |  |  |
| Potential risk of complications related to implant design | 29.62% | 77 | |  |  |  |
| Soft-tissue conditions | 37.31% | 97 | |  |  |  |
| Primary stability | 50.77% | 132 | |  |  |  |
| Aesthetics | 41.54% | 108 | |  |  |  |
| Prosthetic planning | 39.62% | 103 | |  |  |  |
| None | 9.62% | 25 | |  |  |  |
| Other (please specify):  patient expectations  not practicing implantology  there is only one implant system; all others are inadequate  the preference of the referring practice, or refusal on my part if the implant procedure exceeds my surgical competence  referrer preference  navigation  with a conical design, better primary stability is achieved when ridge height is low (1–3 mm), particularly in the maxilla; the narrow implant apex protects the nerve, adjacent roots, and buccal bone while providing similar load capacity compared to wider cylindrical implants  referrer preference  referrer | 3.46% | 9 | |  |  |  |
|  | Answered | 260 | |  |  |  |
|  | Skipped | 16 | |  |  |  |
|  |  |  | |  |  |  |
| 12. Do you use diameter-reduced implants (<3.0 mm)? (multiple answers possible) |  |  | |  |  |  |
| Answer choices | Responses | | |  | | |
| YES, in the anterior region where space is limited | 42.80% | 110 | |  |  |  |
| YES, to avoid augmentation | 5.84% | 15 | |  |  |  |
| YES, in a narrow alveolar ridge | 10.51% | 27 | |  |  |  |
| YES, for minimally invasive treatment in older patients | 19.84% | 51 | |  |  |  |
| NO | 42.80% | 110 | |  |  |  |
| Other (please specify):  if at all possible, then not  mandibular incisors and maxillary lateral incisors  for the maxillary lateral incisors  not practicing implantology  3.25 mm as the smallest diameter  yes, in the anterior region (lateral incisors) when space is limited  mini-implants for edentulous mandible  as temporary mini-implants for interim prostheses  in the mandibular anterior region  2.9 mm only in regions 12, 22, 31, 32, 41, 42  e.g. for four interforaminal implants in cases of narrow bone | 4.28% | 11 | |  |  |  |
|  | Answered | 257 | |  |  |  |
|  | Skipped | 19 | |  |  |  |
|  |  |  | |  |  |  |
| 13. Do you use short implants (<8 mm)? (multiple answers possible) |  |  | |  |  |  |
| Answer choices | Responses | | |  | | |
| YES, in cases of reduced vertical bone height | 59.07% | 153 | |  |  |  |
| YES, to avoid augmentation | 31.66% | 82 | |  |  |  |
| YES, particularly in the posterior mandible | 35.52% | 92 | |  |  |  |
| YES, particularly in the posterior maxilla | 12.74% | 33 | |  |  |  |
| YES, for minimally invasive treatment in older patients | 21.24% | 55 | |  |  |  |
| NO | 24.32% | 63 | |  |  |  |
| Other (please specify):  in combination with additional/longer implants  extremely rare, as a supporting implant  not practicing implantology  very rarely, Straumann WN 6 mm in the posterior region  splinted  epithesis  “minimally invasive” is the next meaningless buzzword after “immediate”; based on experience, even single-tooth implants have resulted in fatalities  not yet, but planned  in high-risk patients to avoid augmentation (in cases of bisphosphonate therapy or after radiotherapy)  exception  or bisphosphonates and bone deficit  at the patient’s request, for example to avoid an extended surgical procedure  very rarely in the posterior mandible  very rarely  rarely, only in specially selected cases  when augmentation cannot be justified, depending on prosthetic planning | 6.18% | 16 | |  |  |  |
|  | Answered | 259 | |  |  |  |
|  | Skipped | 17 | |  |  |  |
|  |  |  | |  |  |  |
| 14. Which imaging modalities do you prefer for implant planning? (multiple answers possible) |  | | |  | |  |
| Answer choices | Responses | | |  | | |
| Periapical radiograph | 10.42% | 27 | |  |  |  |
| Panoramic radiograph | 68.34% | 177 | |  |  |  |
| Paranasal sinus radiograph | 0.39% | 1 | |  |  |  |
| Lateral cephalometric radiograph | 0.39% | 1 | |  |  |  |
| CBCT (cone-beam computed tomography) | 88.42% | 229 | |  |  |  |
| CT | 3.86% | 10 | |  |  |  |
| MRI | 0.39% | 1 | |  |  |  |
| Intraoral scanner | 19.69% | 51 | |  |  |  |
| Facial scanner | 0.77% | 2 | |  |  |  |
| None | 0.39% | 1 | |  |  |  |
| Other (please specify):  not practicing implantology | 0.39% | 1 | |  |  |  |
|  | Answered | 259 | |  |  |  |
|  | Skipped | 17 | |  |  |  |
|  |  |  | |  |  |  |
| 15. Do you use virtual planning prior to implant surgery (even if only occasionally)? |  |  | |  |  |  |
| Answer choices | Responses | | |  | | |
| YES (even if only occasionally) | 73.08% | 190 | |  |  |  |
| NO | 26.92% | 70 | |  |  |  |
| Other (please specify): | 0.00% | 0 | |  |  |  |
|  | Answered | 260 | |  |  |  |
|  | Skipped | 16 | |  |  |  |
|  |  |  | |  |  |  |
| 16. In which cases and how often do you use virtual planning prior to implant surgery? (multiple answers possible) |  | | |  | |  |
| Answer choices | Responses | | |  | | |
| Routinely for all implant placements | 25.27% | 47 | |  |  |  |
| Routinely for most implant placements | 22.04% | 41 | |  |  |  |
| Only in complex cases (e.g. limited bone height/width, challenging anatomy, required parallelism) | 44.09% | 82 | |  |  |  |
| Already during augmentation prior to subsequent implant placement | 15.05% | 28 | |  |  |  |
| When implant placement is simultaneous with augmentation | 17.20% | 32 | |  |  |  |
| In aesthetically demanding cases (e.g. anterior region) | 28.49% | 53 | |  |  |  |
| Only in exceptional cases | 16.67% | 31 | |  |  |  |
| Never | 0.00% | 0 | |  |  |  |
| NO | 0.00% | 0 | |  |  |  |
| Other (please specify):  .  currently being established; thereafter in as many cases as possible | 1.08% | 2 | |  |  |  |
|  | Answered | 186 | |  |  |  |
|  | Skipped | 90 | |  |  |  |
|  |  |  | |  |  |  |
| 17. Who performs the virtual planning? (multiple answers possible) |  |  | |  |  |  |
| Answer choices | Responses | | |  | | |
| Yourself | 89.30% | 167 | |  |  |  |
| Resident / assistant dentist | 10.16% | 19 | |  |  |  |
| Dental technician | 17.65% | 33 | |  |  |  |
| Dental auxiliary staff | 0.00% | 0 | |  |  |  |
| Template manufacturer / external planning service | 12.83% | 24 | |  |  |  |
| Referring colleague (dentist / orthodontist, etc.) | 4.28% | 8 | |  |  |  |
| Other (please specify):  together with a dental technician in a TeamViewer session  joint planning  staff from the prosthodontics department and our department  together with colleagues from prosthodontics | 2.14% | 4 | |  |  |  |
|  | Answered | 187 | |  |  |  |
|  | Skipped | 89 | |  |  |  |
|  |  |  | |  |  |  |
| 18. Why do you not use virtual planning? (multiple answers possible) |  |  | |  |  |  |
| Answer choices | Responses | | |  | | |
| No experience / expertise | 35.71% | 25 | |  |  |  |
| Negative experiences | 5.71% | 4 | |  |  |  |
| Lack of benefit | 48.57% | 34 | |  |  |  |
| No demand from referrers and/or patients | 41.43% | 29 | |  |  |  |
| Insufficient personnel or structural resources | 11.43% | 8 | |  |  |  |
| Time requirement too high | 55.71% | 39 | |  |  |  |
| Costs too high | 61.43% | 43 | |  |  |  |
| Other (please specify):  not practicing implantology  I do not place implants | 2.86% | 2 | |  |  |  |
|  | Answered | 70 | |  |  |  |
|  | Skipped | 206 | |  |  |  |
|  |  |  | |  |  |  |
| 19. Do you use guided surgery (template-assisted surgery) for your implant procedures? |  | | |  | |  |
| Answer choices | Responses | | |  | | |
| YES (even if only occasionally) | 66.54% | 171 | |  |  |  |
| NO | 33.46% | 86 | |  |  |  |
| Other (please specify) | 0.00% | 0 | |  |  |  |
|  | Answered | 257 | |  |  |  |
|  | Skipped | 19 | |  |  |  |
|  |  |  | |  |  |  |
| 20. How often do you use guided surgery (template-assisted surgery) for your implant procedures? (multiple answers possible) |  | | |  | |  |
| Answer choices | Responses | | |  | | |
| Routinely for all implant placements | 12.35% | 21 | |  |  |  |
| Routinely for most implant placements | 17.06% | 29 | |  |  |  |
| Only in complex cases (e.g. limited bone height/width, challenging anatomy, required parallelism) | 55.29% | 94 | |  |  |  |
| Already during augmentation prior to subsequent implant placement | 2.94% | 5 | |  |  |  |
| When implant placement is simultaneous with augmentation | 10.00% | 17 | |  |  |  |
| In aesthetically demanding cases (e.g. anterior region) | 21.76% | 37 | |  |  |  |
| Only in exceptional cases | 25.88% | 44 | |  |  |  |
| Never | 0.00% | 0 | |  |  |  |
| Other (please specify):  single-tooth gap: no; otherwise always a positioning drill guide. Fully guided approximately 25 times about 15 years ago; due to limited mouth opening, it is more suitable for multiple implants in the anterior region. My results with a fully guided technique are nearly comparable and do not justify the additional effort (costs + planning + time) at all (I am past the marketing and ego phase :) )  previously very frequent  edentulous maxilla | 1.76% | 3 | |  |  |  |
|  | Answered | 170 | |  |  |  |
|  | Skipped | 106 | |  |  |  |
|  |  |  | |  |  |  |
| 21. Who manufactures the surgical guides, and how? (multiple answers possible) |  |  | |  |  |  |
| Answer choices | Responses | | |  | | |
| Printed in-house / in the practice | 21.89% | 37 | |  |  |  |
| Milled in-house / in the practice | 3.55% | 6 | |  |  |  |
| Conventionally fabricated in-house / in the practice | 8.88% | 15 | |  |  |  |
| External dental laboratory | 68.05% | 115 | |  |  |  |
| External service provider (e.g. Dedicam / Magelan) | 22.49% | 38 | |  |  |  |
|  | Answered | 169 | |  |  |  |
|  | Skipped | 107 | |  |  |  |
|  |  |  | |  |  |  |
| 22. Why do you not use guided surgery? (multiple answers possible) |  |  | |  |  |  |
| Answer choices | Responses | | |  | | |
| No experience / expertise | 27.91% | 24 | |  |  |  |
| Negative experiences | 5.81% | 5 | |  |  |  |
| Lack of benefit | 54.65% | 47 | |  |  |  |
| No demand from referrers and/or patients | 26.74% | 23 | |  |  |  |
| Insufficient personnel or structural resources | 6.98% | 6 | |  |  |  |
| Time requirement too high | 40.70% | 35 | |  |  |  |
| Costs too high | 59.30% | 51 | |  |  |  |
| Problems with reimbursement by insurance providers | 6.98% | 6 | |  |  |  |
| Other (please specify):  not practicing implantology  I do not place implants  own good surgical experience / skills  practice-owner dependent, planned for the future  complex cases are operated by two surgeons; the clinical situation sometimes differs from the radiographic situation, and changes are not possible with a surgical guide  not helpful in cases of ridge splitting (frequent)  sometimes useful for profiling drilling, if needed  not necessary for an experienced surgeon, rather disadvantageous  I am capable of doing this and I am not a money-driven dentist | 10.47% | 9 | |  |  |  |
|  | Answered | 86 | |  |  |  |
|  | Skipped | 190 | |  |  |  |
|  |  |  | |  |  |  |
| 23. Which methods do you use to assess implant stability and/or osseointegration prior to prosthetic restoration? (multiple answers possible) |  |  | |  |  |  |
| Answer choices |  | | |  | |  |
| Manual assessment / inspection | Responses | | |  | | |
| Insertion torque measurement | 77.69% | 195 | |  |  |  |
| Percussion test | 39.04% | 98 | |  |  |  |
| Resonance frequency analysis (e.g. Osstell / Neotell) | 51.39% | 129 | |  |  |  |
| Periapical radiograph | 15.14% | 38 | |  |  |  |
| Panoramic radiograph (OPG) | 36.65% | 92 | |  |  |  |
| CBCT | 58.96% | 148 | |  |  |  |
| None | 5.18% | 13 | |  |  |  |
| Other (please specify):  what would be the consequence of each reduced measurement value?  not practicing implantology  Periotest  generous healing time provides maximum safety, attachment of crestal bone to the implant, manual insertion of the healing abutment  Periotest  experience and sufficient healing time  peri-implant mucosa free of inflammation | 2.79% | 7 | |  |  |  |
|  | Answered | 251 | |  |  |  |
|  | Skipped | 25 | |  |  |  |
|  |  |  | |  |  |  |
| 24. Which form of implant healing do you prefer (if choice is possible based on stability and other conditions)? |  | | |  | |  |
| Answer choices | Responses | | |  | | |
| Closed (submerged) healing | 83.53% | 208 | |  |  |  |
| Open (non-submerged) healing | 16.47% | 41 | |  |  |  |
|  | Answered | 249 | |  |  |  |
|  | Skipped | 27 | |  |  |  |
|  |  |  | |  |  |  |
| 25. For what reasons or in which scenarios would you prefer closed (submerged) implant healing? (multiple answers possible) |  | | |  | |  |
| Answer choices | Responses | | |  | | |
| Improved wound healing through primary wound closure | 68.80% | 172 | |  |  |  |
| Lower risk of postoperative infection | 65.60% | 164 | |  |  |  |
| Less mechanical loading of the implant during the healing phase | 60.80% | 152 | |  |  |  |
| Financial reasons (second-stage surgery) | 2.80% | 7 | |  |  |  |
| Simultaneous possibility of augmentation procedures | 74.80% | 187 | |  |  |  |
| In patients with an increased risk of complications (e.g. smoking, diabetes, immunosuppression, etc.) | 64.80% | 162 | |  |  |  |
| Following prior augmentation | 35.60% | 89 | |  |  |  |
| None | 1.20% | 3 | |  |  |  |
| Other (please specify):  not practicing implantology  used exclusively, except in cases of immediate implant placement  positive clinical experience  mucosa-supported prosthesis  to protect the bone graft (approximately 80% of cases); additionally, subcrestal implant placement of approximately 0.5 mm reduces risk and is future-oriented  increased soft tissue volume for uncovering  reduced primary stability  superior long-term aesthetic outcome  extremely rare, for prosthetic reasons only | 3.60% | 9 | |  |  |  |
|  | Answered | 250 | |  |  |  |
|  | Skipped | 26 | |  |  |  |
|  |  |  | |  |  |  |
| 26. For what reasons or in which scenarios would you prefer open (non-submerged) implant healing? (multiple answers possible) |  | | |  | |  |
| Answer choices | Responses | | |  | | |
| Possibility of immediate provisional prosthetic restoration | 39.84% | 100 | |  |  |  |
| Improved soft-tissue management | 40.24% | 101 | |  |  |  |
| Lower risk of postoperative infection | 6.37% | 16 | |  |  |  |
| Avoidance of a second surgical procedure for implant exposure | 51.00% | 128 | |  |  |  |
| Less postoperative swelling | 8.37% | 21 | |  |  |  |
| Earlier access for prosthetic procedures | 19.12% | 48 | |  |  |  |
| Reduction of total treatment time | 27.09% | 68 | |  |  |  |
| Financial reasons (reduction of costs) | 15.14% | 38 | |  |  |  |
| None | 17.93% | 45 | |  |  |  |
| Other (please specify):  in immediate implant placement  All-on-4  not practicing implantology  in immediate implant placement  due to proximity to the nerve, the implant cannot be fully submerged  depending on the course of wound healing within 4 weeks, possibly switching to open healing  financially: uncovering individual implants is hardly cost-covering  referrer preference  aesthetics  in cases of dehiscence, otherwise not  immediate implant placement  after immediate implant placement | 4.78% | 12 | |  |  |  |
|  | Answered | 251 | |  |  |  |
|  | Skipped | 25 | |  |  |  |
|  |  |  | |  |  |  |
| 27. Which imaging modalities do you prefer after implant placement? (multiple answers possible) |  | |  | |  |  |
| Answer choices | Responses | | |  | | |
| Periapical radiograph | 41.43% | 104 | |  |  |  |
| Panoramic radiograph | 94.82% | 238 | |  |  |  |
| Paranasal sinus radiograph | 0.40% | 1 | |  |  |  |
| Lateral cephalometric radiograph | 0.00% | 0 | |  |  |  |
| CBCT | 9.56% | 24 | |  |  |  |
| CT | 0.00% | 0 | |  |  |  |
| MRI | 0.00% | 0 | |  |  |  |
| Intraoral scanner | 3.19% | 8 | |  |  |  |
| Facial scanner | 0.40% | 1 | |  |  |  |
| No imaging | 0.40% | 1 | |  |  |  |
| Other (please specify):  not practicing implantology | 0.40% | 1 | |  |  |  |
|  | Answered | 251 | |  |  |  |
|  | Skipped | 25 | |  |  |  |
|  |  |  | |  |  |  |
| 28. In what percentage of cases do you provide immediate provisional prosthetic restorations after implant placement? |  | | |  | |  |
| Answer Choices | Responses | | |  | | |
| Never | 57.83% | 144 | |  |  |  |
| 1-20% | 38.55% | 96 | |  |  |  |
| 21-40% | 2.41% | 6 | |  |  |  |
| 41-60% | 0.40% | 1 | |  |  |  |
| 61-80% | 0.40% | 1 | |  |  |  |
| 81-100% | 0.40% | 1 | |  |  |  |
|  | Answered | 249 | |  |  |  |
|  | Skipped | 27 | |  |  |  |
|  |  |  | |  |  |  |
| 29. In which cases do you provide immediate provisional prosthetic restorations after implant placement? (multiple answers possible) |  | | |  | |  |
| Answer choices | Responses | | |  | | |
| In immediate implant placement | 29.15% | 72 | |  |  |  |
| In early implant placement | 4.05% | 10 | |  |  |  |
| In late implant placement | 7.29% | 18 | |  |  |  |
| In single-tooth restorations | 11.74% | 29 | |  |  |  |
| In immediately loadable full-arch concepts (e.g. All-on-4) | 11.74% | 29 | |  |  |  |
| Only with good primary stability | 24.29% | 60 | |  |  |  |
| Only in the aesthetic zone | 14.57% | 36 | |  |  |  |
| At the request of the referring dentist | 9.72% | 24 | |  |  |  |
| At the patient’s request | 14.57% | 36 | |  |  |  |
| Never | 53.85% | 133 | |  |  |  |
| Other (please specify):  the prosthetic restoration is performed by the referring dentist  very rarely  not practicing implantology  the prosthetic restoration is carried out by the referring dental practice  we never perform the prosthetic treatment ourselves  I do zero prosthetics as an oral and maxillofacial surgeon; those who do it seem to be motivated by financial gain  the prosthetic treatment is done by the dentist  patient compliance, aesthetics  always, but almost exclusively with removable prostheses | 3.64% | 9 | |  |  |  |
|  | Answered | 247 | |  |  |  |
|  | Skipped | 29 | |  |  |  |
|  |  |  | |  |  |  |
| 30. Do you follow up (recall) the patients in whom you have placed implants? |  |  | |  |  |  |
| Answer choices | Responses | | |  | | |
| YES, selected patients are invited | 27.20% | 68 | |  |  |  |
| YES, all patients are invited | 48.00% | 120 | |  |  |  |
| NO | 24.80% | 62 | |  |  |  |
|  | Answered | 250 | |  |  |  |
|  | Skipped | 26 | |  |  |  |
|  |  |  | |  |  |  |
| 31. Do you personally perform definitive implant-supported prosthetic restorations in addition to implant surgery? |  | | |  | |  |
| Answer choices | Responses | | |  | | |
| YES, always | 4.38% | 11 | |  |  |  |
| YES, occasionally | 24.70% | 62 | |  |  |  |
| NO | 70.92% | 178 | |  |  |  |
|  | Answered | 251 | |  |  |  |
|  | Skipped | 25 | |  |  |  |
|  |  |  | |  |  |  |
| 32. Why do you not personally perform definitive implant-supported prosthetic restorations in addition to implant surgery? (multiple answers possible) |  |  | |  |  |  |
| Answer choices | Responses | | |  | | |
| No experience / expertise | 31.64% | 56 | |  |  |  |
| Negative experiences | 0.56% | 1 | |  |  |  |
| No personal interest | 28.81% | 51 | |  |  |  |
| No financial benefit | 2.26% | 4 | |  |  |  |
| Performed by another colleague in the practice/clinic | 13.56% | 24 | |  |  |  |
| No demand from referrers and/or patients | 26.55% | 47 | |  |  |  |
| Insufficient personnel or structural resources | 9.60% | 17 | |  |  |  |
| Time requirement too high | 5.08% | 9 | |  |  |  |
| Costs too high | 0.00% | 0 | |  |  |  |
| Work in a purely surgical referral practice | 77.40% | 137 | |  |  |  |
| Other (please specify):  not practicing implantology  I do not place implants  because I am an oral and maxillofacial surgeon and have sufficient surgical workload  due to good collaboration with referring dentists  as a department of oral and maxillofacial surgery, we do not provide prosthetic treatment ourselves  prosthodontists can do this better  political reasons – general dentists in rural areas are very sensitive and become upset | 3.95% | 7 | |  |  |  |
|  | Answered | 177 | |  |  |  |
|  | Skipped | 99 | |  |  |  |
|  |  |  | |  |  |  |
|  |  |  | |  |  |  |
| 33. Which preoperative management do you routinely use for implant placement? (multiple answers possible) |  | | |  | |  |
| Answer choices | Responses | | |  | | |
| Pre-emptive analgesia (e.g. ibuprofen) | 15.71% | 11 | |  |  |  |
| Glucocorticoids (e.g. dexamethasone) oral | 1.43% | 1 | |  |  |  |
| Glucocorticoids (e.g. dexamethasone) intravenous | 4.29% | 3 | |  |  |  |
| Antibiotics oral | 72.86% | 51 | |  |  |  |
| Antibiotics intravenous | 10.00% | 7 | |  |  |  |
| Antiseptic rinses (e.g. chlorhexidine) | 81.43% | 57 | |  |  |  |
| Professional dental cleaning | 52.86% | 37 | |  |  |  |
| Optimization of patient compliance | 32.86% | 23 | |  |  |  |
| None of the above | 2.86% | 2 | |  |  |  |
| Other (please specify):  Professional dental cleaning in the referring dental practice  Local administration of glucocorticoids  Professional dental cleaning performed elsewhere | 4.29% | 3 | |  |  |  |
|  | Answered | 70 | |  |  |  |
|  | Skipped | 206 | |  |  |  |
|  |  |  | |  |  |  |
| 34. Which postoperative management do you use after implant placement? (multiple answers possible) |  | |  | |  |  |
| Answer choices | Responses | | |  | | |
| Pain medication as needed | 68.06% | 49 | |  |  |  |
| Pain medication with a medication plan | 27.78% | 20 | |  |  |  |
| Glucocorticoids (e.g. dexamethasone) oral | 4.17% | 3 | |  |  |  |
| Glucocorticoids (e.g. dexamethasone) intravenous | 4.17% | 3 | |  |  |  |
| Antibiotics oral | 76.39% | 55 | |  |  |  |
| Antibiotics intravenous | 2.78% | 2 | |  |  |  |
| Antiseptic rinses (e.g. chlorhexidine) | 59.72% | 43 | |  |  |  |
| Cooling | 76.39% | 55 | |  |  |  |
| Restriction of physical activity / avoidance of sports | 56.94% | 41 | |  |  |  |
| Soft diet / dietary modification | 80.56% | 58 | |  |  |  |
| Temporary avoidance of dairy products | 12.50% | 9 | |  |  |  |
| None | 0.00% | 0 | |  |  |  |
| Other (please specify) | 0.00% | 0 | |  |  |  |
|  | Answered | 72 | |  |  |  |
|  | Skipped | 204 | |  |  |  |
|  |  |  | |  |  |  |
| 35. Which factors, in your opinion, are most likely to negatively affect the long-term success of implant treatment? (multiple answers possible) |  | | |  | |  |
| Answer choices | Responses | | |  | | |
| Implant system used | 19.44% | 14 | |  |  |  |
| Implant design used | 23.61% | 17 | |  |  |  |
| Timing of implant placement | 30.56% | 22 | |  |  |  |
| Positioning of the implant | 66.67% | 48 | |  |  |  |
| Placement in augmented bone | 29.17% | 21 | |  |  |  |
| Surgical technique | 51.39% | 37 | |  |  |  |
| Omission of augmentation | 38.89% | 28 | |  |  |  |
| Limited operator experience | 76.39% | 55 | |  |  |  |
| Poor patient compliance | 76.39% | 55 | |  |  |  |
| Poor oral hygiene | 84.72% | 61 | |  |  |  |
| Patient medications and comorbidities | 62.50% | 45 | |  |  |  |
| Lifestyle factors (e.g. smoking) | 75.00% | 54 | |  |  |  |
| Prosthetic restoration: emergence profile design | 54.17% | 39 | |  |  |  |
| Retention of the prosthetic restoration (screw-retained vs. cemented) | 31.94% | 23 | |  |  |  |
| Aftercare | 33.33% | 24 | |  |  |  |
| None | 0.00% | 0 | |  |  |  |
| Other (please specify):  Complex augmentations; Prosthetic insufficiency  Biological individuality  A key factor for long-term success with implants is the creation and maintenance of attached gingiva  soft-tissue management  soft tissue, (lack of) attached gingiva | 6.94% | 5 | |  |  |  |
|  | Answered | 72 | |  |  |  |
|  | Skipped | 204 | |  |  |  |
|  |  |  | |  |  |  |
| 36. What do you consider to be absolute contraindications for implant placement? (multiple answers possible) |  |  | |  |  |  |
| Answer choices | Responses | | |  | | |
| Systemic underlying diseases (e.g. diabetes mellitus) | 1.41% | 1 | |  |  |  |
| Radiotherapy (<12 months before planned implant placement) | 66.20% | 47 | |  |  |  |
| Radiotherapy (>12 months before planned implant placement) | 14.08% | 10 | |  |  |  |
| Antiresorptives (e.g. low-potency bisphosphonates for osteoporosis) | 9.86% | 7 | |  |  |  |
| Antiresorptives (e.g. high-potency bisphosphonates for breast cancer) | 57.75% | 41 | |  |  |  |
| Antiresorptives (e.g. RANK-L antibodies, e.g. denosumab) | 29.58% | 21 | |  |  |  |
| Medications (e.g. proton pump inhibitors) | 1.41% | 1 | |  |  |  |
| Antidepressants (e.g. SSRIs) | 1.41% | 1 | |  |  |  |
| Poor oral hygiene | 40.85% | 29 | |  |  |  |
| Poor patient compliance | 47.89% | 34 | |  |  |  |
| Lifestyle factors (e.g. smoking) | 4.23% | 3 | |  |  |  |
| None | 9.86% | 7 | |  |  |  |
|  | Answered | 71 | |  |  |  |
|  | Skipped | 205 | |  |  |  |
|  |  |  | |  |  |  |
| 37. Do you use platelet-rich fibrin (PRF) in implant surgery? |  |  | |  |  |  |
| Answer choices | Responses | | |  | | |
| YES, in all cases | 9.72% | 7 | |  |  |  |
| YES, in selected cases | 45.83% | 33 | |  |  |  |
| NO | 44.44% | 32 | |  |  |  |
|  | Answered | 72 | |  |  |  |
|  | Skipped | 204 | |  |  |  |
|  |  |  | |  |  |  |
| 38. For what purposes do you use platelet-rich fibrin (PRF) in implant surgery? (multiple answers possible) |  | |  | |  |  |
| Answer choices | Responses | | |  | | |
| Biological conditioning of the implant surface | 20.11% | 37 | |  |  |  |
| Placement of PRF into the implant site (osteotomy) | 20.65% | 38 | |  |  |  |
| Biological conditioning of biomaterials (e.g. bone substitute material, membranes) | 63.59% | 117 | |  |  |  |
| Injection into the surgical site (e.g. liquid PRF) | 15.76% | 29 | |  |  |  |
| Placement into the surgical site (e.g. PRF membrane) | 58.15% | 107 | |  |  |  |
| Covering of exposed implant surfaces | 18.48% | 34 | |  |  |  |
| Other (please specify):  no PRF  we do not use it  never, too time-consuming  not used  currently not performed  never  not at all  never  not practicing implantology  provides no benefit  I do not use it  never  unnecessary  I do not use it  aesthetics  not at all  augmentation  never  not at all  I do not place implants  never, because honest surgery keeps me sufficiently occupied  billing optimization  no PRF application  never  never  never  not yet used  not used  I never use it  no  improvement of soft tissue healing  never  why bother, outcomes with autogenous bone grafts are good; what is needed is gentle tissue handling and patience; nature does it as well and at lower cost  no PRF  socket preservation  never  not at all  not at all  never  never  I do not use it  no  we do not use it  not used  no  no application  internal sinus lift  not at all  after 5 years I have a success rate of 99% with very good to good aesthetics  hokus-pokus | 27.17% | 50 | |  |  |  |
|  | Answered | 184 | |  |  |  |
|  | Skipped | 92 | |  |  |  |
|  |  |  | |  |  |  |
